# MiR-148b-3p在肺腺癌中的表达及其与患者预后的相关性

**DOI:** 10.3779/j.issn.1009-3419.2019.05.07

**Published:** 2019-05-20

**Authors:** 泽凯 黄, 少雷 李, 媛媛 马, 楠 吴, 跃 杨

**Affiliations:** 100142 北京，北京大学肿瘤医院暨北京市肿瘤防治研究所胸外二科 恶性肿瘤发病机制及转化研究教育部重点实验室 Key Laboratory of Carcinogenesis and Translational Research (Ministry of Education), Department of Thoracic Surgery Ⅱ, Peking University Cancer Hospital and Institute, Beijing 100142, China

**Keywords:** miR-148b-3p, 肺肿瘤, 预后, miR-148b-3p, Lung neoplasms, Prognosis

## Abstract

**背景与目的:**

MiR-148b-3p是一种重要的微小RNA，已经被报道与多种癌症密切相关，但其在肺腺癌中的作用仍不清楚。本研究的目的是检测miR-148b-3p在肺腺癌中的表达水平，并分析其与临床病理特征及患者预后的相关性。

**方法:**

收集2011年1月-2012年12月在本科室经手术切除的肺腺癌患者的肿瘤标本123例，利用实时荧光定量PCR方法检测miR-148b-3p的表达量，分析其与患者临床病理特征的相关性。利用多因素*Cox*比例风险模型分析影响患者总生存的独立预测因子。利用*Kaplan*-*Meier*生存分析方法估计miR-148b-3p高表达组和低表达组患者的总生存期，并使用*Log*-*rank*检验方法进行显著性检验。

**结果:**

在123例肺腺癌患者中，有71例高表达miR-148b-3p，52例低表达。miR-148b-3p与肿瘤的分化程度（*P*=0.001）、肿瘤大小（*P*=0.007）显著相关，而与年龄、性别、吸烟史、饮酒史、脉管癌栓、胸膜侵犯、淋巴结转移、远处转移和术后治疗不存在统计学显著的相关性。多因素*Cox*比例风险模型分析显示肿瘤大小（*P*=0.032）、淋巴结转移（*P*=0.005）和miR-148b-3p表达量（*P*=0.047）是影响患者总生存的独立预测因子。*Kaplan*-*Meier*生存分析显示miR-148b-3p高表达组患者的总生存显著优于miR-148b-3p低表达组患者（*P*=0.010）。

**结论:**

MiR-148b-3p在肺腺癌中与肿瘤的分化程度、肿瘤大小显著相关，并且是影响患者总生存的独立预测因子。MiR-148b-3p高表达组患者的总生存显著优于低表达组患者。因此，miR-148b-3p可能成为新的肺腺癌治疗靶标或预后生物标志物。

## Abstract (en)

**Background and objective:**

MiR-148b-3p is an important microRNA that has been reported to be significantly related to various types of cancer, but its role in lung adenocarcinoma remains elusive. The purpose of this study is to detect the expression level of miR-148b-3p in lung adenocarcinoma specimens, and to analyze its correlation with the clinicopathological features as well as the prognosis of patients with lung adenocarcinoma.

**Methods:**

A total of 123 tumor specimens from lung adenocarcinoma patients who underwent surgical resection in our department from January 2011 to December 2012 were collected. The expression of miR-148b-3p was detected by quantitative real-time PCR (qRT-PCR), and its correlation with clinicopathological features of patients with lung adenocarcinoma was analyzed. Multivariate *Cox* proportional hazard models were used to analyze independent predictors of overall survival in patients with lung adenocarcinoma. The overall survival (OS) of patients in miR-148b-3p high expression group and miR-148b-3p low expression group were estimated by means of the *Kaplan*-*Meier* method and were compared using the *Log*-*rank* test method.

**Results:**

Of the 123 patients with lung adenocarcinoma, 71 were in miR-148b-3p high expression group and 52 in low expression group. MiR-148b-3p was significantly associated with tumor grade (*P*=0.001) and tumor size (*P*=0.007), but not with age, gender, smoking history, history of alcohol, tumor thrombus, pleural invasion, node status or metastasis status. Multivariate *Cox* proportional hazard model analysis showed that tumor size (*P*=0.032), node status (*P*=0.005) and miR-148b-3p expression level (*P*=0.047) were significant independent predictors of overall survival of patients with lung adenocarcinoma. *Kaplan*-*Meier* survival analysis showed that the overall survival of patients with high expression of miR-148b-3p was significantly better than that of patients with low expression (*P*=0.010).

**Conclusion:**

MiR-148b-3p was significantly associated with tumor grade and tumor size in lung adenocarcinoma, and served as an independent predictor of overall survival of patients with lung adenocarcinoma. The overall survival of patients with high expression level of miR-148b-3p was significantly better than that of patients with low expression.

肺癌是全球发病率最高的癌症类型，并且是癌症死亡的主要原因。据估计2018年全球将有210万新发肺癌病例和180万新发死亡病例，死亡人数占癌症总体死亡人数的18.4%^[1]^。在中国，肺癌也是最常见的癌症类型和癌症死亡的主要原因。目前最新的文献显示2015年中国新发肺癌病例为73.3万例，肺癌死亡病例为61.0万例^[2]^。肺腺癌是最常见的肺癌亚型，也是非吸烟肺癌患者中最常见的肺癌组织学类型^[3]^。尽管近些年来由于靶向治疗等新的治疗方式的飞速发展使得肺癌的治疗取得重大进展，但是由于约70%的患者在诊断为肺癌时就已经出现远处转移或为局部晚期，肺癌在美国的总体生存率仍只有18%左右^[4]^。因此，寻找新的肺癌治疗靶标和预后生物标志物显得尤为重要。

微小RNA（microRNA, miRNA）是一类含有约22个核苷酸的单链小非编码RNA，其通过介导翻译抑制或促进靶mRNA的降解而作为内源基因调节剂发挥重要作用^[5]^。据估计微小RNA可以靶向超过30%的人类基因组，而且微小RNA的异常表达已经被证实与多种癌症的发生密切相关^[6]^。MiR-148b-3p作为一种重要的微小RNA，已经被报道与肾癌、胃癌、乳腺癌等多种癌症密切相关，但与肺腺癌的关系尚不清楚^[7-9]^。本研究通过检测miR-148b-3p在肺腺癌中的表达水平，分析其与临床病理特征及患者预后的相关性。

## 1 材料与方法

### 1.1 研究对象及临床资料

收集2011年1月-2012年12月之间在我科进行手术切除的肺腺癌患者的肿瘤标本123例。纳入本研究的患者均为初诊且术前未行放化疗等抗肿瘤治疗，排除伴其他恶性肿瘤或有既往恶性肿瘤病史的患者。本项研究通过了本院伦理委员会批准并取得患者的知情同意。在123例肺腺癌患者中男性73例，女性50例。年龄范围为31岁-86岁，其中≥60岁56例， < 60岁67例。肿瘤分化程度为中高分化（G1-2）的肿瘤标本75例，低分化（G3-4）48例。肿瘤大小≥3 cm63例， < 3 cm60例。存在淋巴结转移（node status positive）63例，不存在淋巴结转移（node status negative）60例。存在远处转移（metastasis status positive）11例，不存在远处转移（metastasis status negative）112例。未接受术后治疗56例；而接收术后治疗67例（其中接收单纯化疗46例，接收单纯放疗1例，接收单纯靶向治疗5例；同时接收放化疗10例，同时接收放化疗和靶向治疗2例，同时接收化疗和靶向治疗2例，同时接收放疗和靶向治疗1例）。

### 1.2 MiR-148b-3p表达量的测定及分组

病理组织保存于-80 ℃冰箱中，按照Trizol试剂（Invitrogen, Carlsbad, CA, USA）说明书的方法提取肺腺癌肿瘤组织的总RNA，并使用NanoDrop机器进行RNA定量与质检。本研究使用加尾法逆转录microRNA为cDNA。首先使用polyA聚合酶（New England Biolabs, USA）将polyA尾添加到0.5 μg总RNA中，然后使用M-MLV cDNA第一链逆转录试剂盒（Cat No. C28025032, Invitrogen）将microRNA逆转录为cDNA。使用SYBR Green实时荧光定量PCR方法检测miR-148b-3p的表达量，并以U6为内参基因，通过2^-△△Ct^法计算倍数变化。实时荧光定量PCR所用的miR-148b-3p上游引物为：5’-TCAGTGCATCACAGAACTTTGT-3’，下游引物为：5’-GCGAGCACAGAATTAATACGAC-3’。U6的上游引物为：5’-CTCGCTTCGGCAGCACA-3’，下游引物为5’-AACGCTTCACGAATTTGCGT-3’。此外，根据miR-148b-3p的相对表达量，借助Cutoff Finder程序^[10]^确定MiR-148b-3p分组的最佳分界值，该程序可基于对数秩检验（*Log*-*rank* test）计算最佳分界值。

### 1.3 随访

通过电话、邮件、复查等方式进行随访，末次随访时间为2018年7月12日。在术后的前两年，患者每3个月随访一次；术后3年-5年每6个月随访一次；术后5年以后，每年随访一次。对有症状的患者给予相应的检查。在随访期间，常规的复查项目包括详细的病史询问、体格检查、胸部CT检查等。患者术后的不良反应、疾病复发、死亡等都被详细记录。

### 1.4 统计学分析

采用IBM SPSS version 24（Chicago, USA）软件进行统计学分析。采用*χ*^2^检验或*Fisher*精确概率法对两组计数资料进行比较。总生存期（overall survival, OS）定义为患者从接受手术到死亡或末次随访的时间，采用*Kaplan*-*Meier*生存分析方法进行估计并且采用*Log*-*rank*检验方法进行显著性检验。检验水准*α*=0.05。利用单因素和多因素*Cox*比例风险模型分析影响患者总生存的独立预测因子。多因素*Cox*比例风险模型纳入的变量为单因素*Cox*分析中有统计学意义（*P* < 0.100）的变量。

## 2 结果

### 2.1 MiR-148b-3p的相对表达量与肺腺癌患者临床病理特征的相关性

根据miR-148b-3p的相对表达量，借助Cutoff Finder程序^[10]^将123例肺腺癌患者分为71例miR-148b-3p高表达组和52例miR-148b-3p低表达组。MiR-148b-3p表达量与肿瘤的分化程度（*P*=0.001）、肿瘤大小（*P*=0.007）显著相关，而与年龄、性别、吸烟史、饮酒史、脉管癌栓、胸膜侵犯、淋巴结转移和远处转移不存在统计学显著的相关性，见表 1。

**1:** miR-148b-3p的表达量与肺腺癌患者临床病理特征的相关性 Correlation between expression level of miR-148b-3p and clinicopathological features of patients with lung adenocarcinoma

Variable	miR-148b-3p expression level		*χ*^2^	*P*
	High	Low		
Age (yr)				0.539
≥60	34	22	0.377	
< 60	37	30		
Gender				0.995
Male	41	32	< 0.001	
Female	30	20		
Smoking history				0.817
No	37	26	0.054	
Yes	34	26		
History of alcohol				0.887
No	50	36	0.020	
Yes	21	16		
Tumor grade				0.001
G_1-2_	52	23	10.615	
G_3-4_	19	29		
Tumor thrombus				0.715
Negative	54	41	0.133	
Positive	17	11		
Pleural invasion				0.698
Negative	27	18	0.151	
Positive	44	34		
Tumor size (cm)				0.007
≥3	29	34	7.234	
< 3	42	18		
Node status				0.817
No	34	26	0.054	
Yes	37	26		
Metastasis status				0.133
0	67	45	2.259	
1	4	7		
Adjuvant therapy				0.805
Negative	33	23	0.061	
Positive	38	29		

### 2.2 MiR-148b-3p表达量与肺腺癌患者总生存之间关系的*Kaplan*-*Meier*生存分析

所有患者的中位随访时间是68.0个月（95%CI: 62.9-73.1）。MiR-148b-3p高表达组的中位总生存期未达到，其1年、3年、5年的估计总生存率分别是97.2%、79.8%、69.4%。而miR-148b-3p低表达组的中位总生存期是51.0个月（95%CI: 36.7-65.3），其1年、3年、5年的估计总生存率分别是92.3%、67.3%、43.1%。*Kaplan*-*Meier*生存曲线图分析显示miR-148b-3p高表达组患者的总生存时间显著优于低表达组患者（*P*=0.010），见图 1。

**1:**
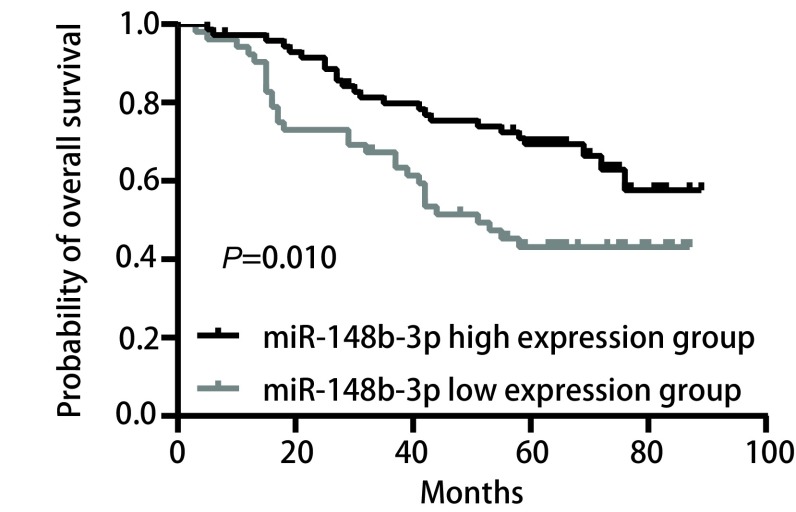
miR-148b-3p表达量与肺腺癌患者总生存的*Kaplan*-*Meier*生存曲线分析图 *Kaplan*-*Meier* survival analysis between miR-148b-3p expression level and overall survival of patients with lung adenocarcinoma

### 2.3 肺腺癌患者总生存的单因素和多因素分析

多因素*Cox*比例风险模型分析显示肿瘤大小（*P*=0.032）、淋巴结转移（*P*=0.005）和miR-148b-3p表达量（*P*=0.047）是影响肺腺癌患者总生存的独立预测因子，见表 2。

**2:** 肺腺癌患者总生存的单因素和多因素*Cox*比例风险模型分析 Univariate and multivariate *Cox* proportional hazard models analysis of overall survival of patients with lung adenocarcinoma

Variable	Univariate analysis			Multivariate analysis	
	HR (95%CI)	*P*		aHR (95%CI)	*P*
Age (yr)		0.036			0.649
≥60	Reference			Reference	
< 60	1.824 (1.040-3.200)			1.153 (0.624-2.130)	
Gender		0.991			
Male	Reference				
Female	1.003 (0.583-1.728)				
Smoking history		0.844			
No	Reference				
Yes	1.056 (0.616-1.810)				
History of alcohol		0.430			
No	Reference				
Yes	0.782 (0.424-1.442)				
Tumor grade		0.042			0.487
G_1-2_	Reference			Reference	
G_3-4_	1.751 (1.021-3.003)			1.225 (0.691-2.171)	
Tumor thrombus		0.052			0.216
Negative	Reference			Reference	
Positive	1.79 (0.995-3.221)			1.483 (0.794-2.769)	
Pleural invasion		0.304			
Negative	Reference				
Positive	0.751 (0.434-1.298)				
Tumor size (cm)		0.004			0.032
≥3	Reference			Reference	
< 3	0.431 (0.244-0.762)			0.526 (0.292-0.947)	
Node status		0.004			0.005
No	Reference			Reference	
Yes	2.308 (1.305-4.082)			2.255 (1.272-3.998)	
Metastasis status		0.062			0.142
0	Reference			Reference	
1	2.147(0.963-4.783)			1.827 (0.817-4.090)	
Adjuvant therapy		0.032			0.744
Negative	Reference			Reference	
Positive	1.881 (1.056-3.352)			1.114 (0.584-2.125)	
miR-148b-3p expression level		0.012			0.047
High	Reference			Reference	
Low	2.012 (1.170-3.460)			1.762 (1.007-3.082)	
HR: hazard ratio; aHR: adjusted hazard ratio.					

## 3 讨论

在美国，癌症的总体5年相对存活率超过60%，而肺癌的5年相对存活率只有18%左右^[4]^。当肿瘤局限于肺部且只在局部淋巴结扩散的时候，最有效的治疗方法是手术。但是大多数肺癌患者在初次诊断时就已经出现远处转移或被为局部晚期，并不符合手术切除的条件，这是肺癌总体预后不良的一个重要原因^[11]^。因此，关于寻找新的肺癌治疗靶标或预后生物标志物的研究，对于提高肺癌患者总体生存率和改善其预后，将具有十分重要的意义。

微小RNA（miRNA）是一类非编码RNA分子，通过与互补靶mRNA结合导致mRNA翻译抑制或降解，在细胞分化、增殖和存活中发挥重要作用^[12]^。在1993年，第一个微小RNA在秀丽隐杆线虫上被发现^[13]^。7年后，第一个哺乳动物微小RNA，即let-7，也被发现^[14]^。这两个关键事件导致了之后的一系列基因组研究，揭示了许多miRNA和其他非编码RNA的广泛转录^[15]^。近些年来，微小RNA已成为癌症研究的一个热点。许多研究报道微小RNA可以通过控制其靶mRNA的表达以促进或抑制肿瘤生长、侵袭、血管生成等，证明了微小RNA在癌症生物学中发挥着重要的功能^[16]^。此外，据报道肿瘤的微小RNA表达谱可以用来定义相关的亚型，预测患者存活率和治疗反应^[6]^。同时，由于微小RNA在临床样本中的有着比mRNA更高的稳定性，因此比mRNA更加适合作为预后指标^[17]^。

MiR-148b-3p作为一种重要的miRNA，已经被报道与多种癌症密切相关。Zhang等^[7]^基于GEO分析，发现肾癌中miR-148b-3p的表达降低，并且通过功能实验证明了miR-148b-3p可以通过调节FGF2-FGFR2信号通路，从而促进肾癌细胞凋亡并抑制细胞增殖、迁移和肿瘤生长。Li及其同事^[9]^发现miR-148b-3p的表达与中的胃癌Dock6（一种非典型Rho鸟嘌呤核苷酸交换因子）的表达呈负相关，并通过抑制Dock6/Rac1/Cdc42轴降低胃癌细胞的运动性，从而抑制胃癌转移。同时，miR-148b-3p高表达组的胃癌患者的预后显著优于低表达组的患者。然而，目前miR-148b-3p在肺癌中的作用仍不清楚。

在本研究中，我们收集了2011年1月-2012年12月之间在我科进行手术切除的肺腺癌肿瘤标本123例。我们发现miR-148b-3p在肺腺癌肿瘤组织中的表达量与肿瘤分化程度（*P*=0.001）、肿瘤大小（*P*=0.007）显著相关。我们通过单因素*Cox*比例风险模型分析发现，年龄（*P*=0.036）、肿瘤分化程度（*P*=0.042）、脉管癌栓（*P*=0.052）、肿瘤大小（*P*=0.004）、淋巴结转移（*P*=0.004）、远处转移（*P*=0.062）、术后治疗（*P*=0.032）和miR-148b-3p表达水平（*P*=0.012）与患者的总生存相关。进一步将这些变量纳入多因素*Cox*比例风险模型进行分析，发现miR-148b-3p表达量（*P*=0.047）、肿瘤大小（*P*=0.032）、淋巴结转移（*P*=0.005）是影响患者总生存的独立预测因子。同时，*Kaplan*-*Meier*生存分析显示miR-148b-3p高表达组患者的总生存显著优于miR-148b-3p低表达组患者（*P*=0.010），表明miR-148b-3p在肺腺癌中可能发挥着抑癌基因的作用。但是，miR-148b-3p在肺腺癌中具体的生物学作用和机制尚不清楚，亟需进一步阐明。如果miR-148b-3p的生物学作用能在体内外实验中得到进一步验证，那么其将有助于肺腺癌的检测与治疗。本研究由于是回顾性分析，且样本量较少（123例），无法充分控制混杂因素的影响，尚不足以指导临床实践，但却为后续的临床和基础研究提供了有意义的参考。

综上所述，miR-148b-3p表达量与肺腺癌的肿瘤分化程度、肿瘤大小显著相关，是影响患者总生存的独立预测因子。同时，miR-148b-3p高表达组患者的总生存显著优于miR-148b-3p低表达组患者。因此，miR-148b-3p可能在肺腺癌中扮演了非常重要的角色，有望成为新的肺腺癌治疗靶标或预后生物标志物。
